# Selective Laser Trabeculoplasty after a Previous Glaucoma Treatment

**DOI:** 10.3390/biomedicines12102212

**Published:** 2024-09-27

**Authors:** Patrick Thelen, Daniel Böhringer, Philip Keye, Thomas Reinhard, Jan Lübke

**Affiliations:** Department of Ophthalmology, University Medical Center Freiburg, Faculty of Medicine, Albert Ludwig University of Freiburg, 79106 Freiburg, Germanyjan.luebke@uniklinik-freiburg.de (J.L.)

**Keywords:** glaucoma, selective laser trabeculoplasty, pressure-reducing procedure, open-angle glaucoma

## Abstract

Background/Objectives: Recent prospective studies have shown that selective laser tra-beculoplasty (SLT) is a safe and cost-effective alternative to pressure-reducing eye drop therapy as a first-line treatment for ocular hypertension or open-angle glaucoma. In addition to its comparable efficacy to eye drop therapy, SLT has been particularly effective in delaying the time until a surgical intervention is needed. The aim of our evaluation is to analyze patients who have received SLT following a pressure-reducing procedure. The primary endpoint is the duration until a subsequent interventional or surgical procedure is required. Methods: A retrospective analysis of 98 patients who underwent selective laser trabeculoplasty following a previous pressure-reducing procedure between 2017 and 2023. The statistical analyses included Cox regression and Kaplan–Meier survival estimations. Results: In total, 122 eyes of 98 patients received selective laser trabeculoplasty following a previous pressure-reducing procedure at the Department of Ophthalmology in Freiburg. The median follow-up period was 381.5 days (range 43.25–862.75 days). Approximately 68% of the eyes did not require another pressure-reducing procedure 365 days after the intervention, while about 58% of the eyes remained without another procedure after 730 days, according to Kaplan–Meier analysis. No significant difference was found between the different types of glaucoma regarding the duration until a subsequent pressure-reducing procedure was needed. The study indicated a tendency for patients with pseudoexfoliation glaucoma to undergo a secondary intervention earlier compared to those with primary open-angle glaucoma (*p* = 0.16). The intraocular pressure before SLT had a significant impact on the duration until the subsequent operation (*p* = 0.005). Conclusions: SLT is an effective method even after a previous pressure-reducing procedure for patients in whom further pressure-reducing interventions need to be delayed.

## 1. Introduction

Selective laser trabeculoplasty (SLT) is a minimally invasive treatment method, particularly for patients with open-angle glaucoma or ocular hypertension [1]. Numerous studies have demonstrated the effectiveness of SLT in lowering intraocular pressure (IOP), which is a significant risk factor for the progression of glaucoma. It has been shown that SLT effectively reduces intraocular pressure by approximately 20–30% in surgery-naive eyes, comparable to the reduction achieved with conventional topical medications [2].

The exact mechanism of action of SLT is not fully understood yet. According to current knowledge, SLT primarily reduces intraocular pressure through mechanical and cellular effects [3,4]. This process involves the coagulative damage of the cells within the trabecular meshwork, leading to collagen contraction and scarring, which tightens the trabecular meshwork. This tightening opens the adjacent untreated intertrabecular spaces, enhancing aqueous humor outflow. Additionally, the disruption of pigment granules triggers the migration of macrophages and subsequent phagocytosis, which helps clean the trabecular meshwork. However, the coagulative effect does not seem to be essential for lowering intraocular pressure [4,5,6].

SLT is considered a safe procedure with a minimal risk of complications. Side effects may include temporary discomfort such as mild inflammation and a temporary increase in intraocular pressure immediately after the procedure. Serious complications like persistent iritis or mechanical damage to surrounding eye structures are extremely rare [1]. While SLT can effectively lower intraocular pressure in the short and medium term, its long-term effectiveness remains the subject of ongoing research. According to current research, the initial pressure-lowering effect tends to diminish over time, potentially necessitating retreatment or additional therapy. However, Gazzard et al. demonstrated that compared to medical pressure-lowering therapy, SLT allows for better long-term glaucoma control, with a reduced number of glaucoma and cataract surgeries [1]. Furthermore, Philippin et al. demonstrated a superior effect of SLT compared to timolol eye drops [7]. Studies comparing selective laser trabeculoplasty with topical pressure-lowering eye drops predominantly evaluate cohorts of therapy-naïve eyes.

The European Glaucoma Society and the American Academy of Ophthalmology recommend SLT as an initial treatment option for open-angle glaucoma (OAG) and ocular hypertension (OHT) [8,9]. SLT can be particularly beneficial for patients who prefer a non-invasive treatment option, those in whom the time until surgery needs to be delayed, or those experiencing side effects from local or systemic medication therapy [10].

There are few studies regarding the outcomes of patients who underwent SLT after glaucoma surgery. However, these studies have shown that SLT can effectively lower intraocular pressure in patients who have previously undergone filtering glaucoma surgery [2,11,12].

The aim of our study is to evaluate patients who underwent SLT following a prior glaucoma surgical procedure. The primary endpoint chosen is the duration until a subsequent glaucoma surgery.

## 2. Materials and Methods

We conducted a retrospective monocentric analysis of all patients who underwent selective laser trabeculoplasty following a prior pressure-reducing procedure (see Table 1) at the Eye Center, University Medical Center Freiburg, between 2017 and 2023. We included only those patients for whom an additional intervention would have been indicated following a prior pressure-reducing procedure, with decisions made individually for each patient. The decision was made based on the individual IOP trend, OCT diagnostics, and the progression of visual field loss, either during the inpatient stay or as part of the outpatient follow-up.

Initially, a database search was conducted to identify all patients within the specified period who underwent a pressure-reducing surgery prior to undergoing SLT at our institution. The data presented in the results section were manually extracted for each patient individually. Statistical analysis of the collected data was performed using the open-source statistical program R version 2022.02.2 (https://www.r-project.org, accessed 6 June 2024). The graphical representations, including all charts and diagrams, were also generated using R. The results were analyzed descriptively, and frequency distributions were reported in percentages. The collected data were represented using the mean (including 95% confidence interval) and median (including quartiles). The significance level was set at 5% for all tests. Thus, a *p*-value ≤ 0.05 was considered statistically significant. The dependencies of individual factors were calculated using Cox regression. The calculation and presentation of survival rates were performed using Kaplan–Meier analysis.

## 3. Results

In the stated period, SLT following a prior pressure-reducing procedure was performed on 122 eyes of 98 patients. Table 2 shows the various collected characteristics of the patient cohort. The intraocular pressure after the SLT was measured in 38 eyes before a subsequent intervention. The number of pressure-lowering medications after the SLT was recorded in 45 eyes prior to further treatment. For all other patients, no consultation for intraocular pressure measurement took place at our clinic before any additional glaucoma treatment. The type of glaucoma for all eyes is shown in Table 3.

**Figure 1:**
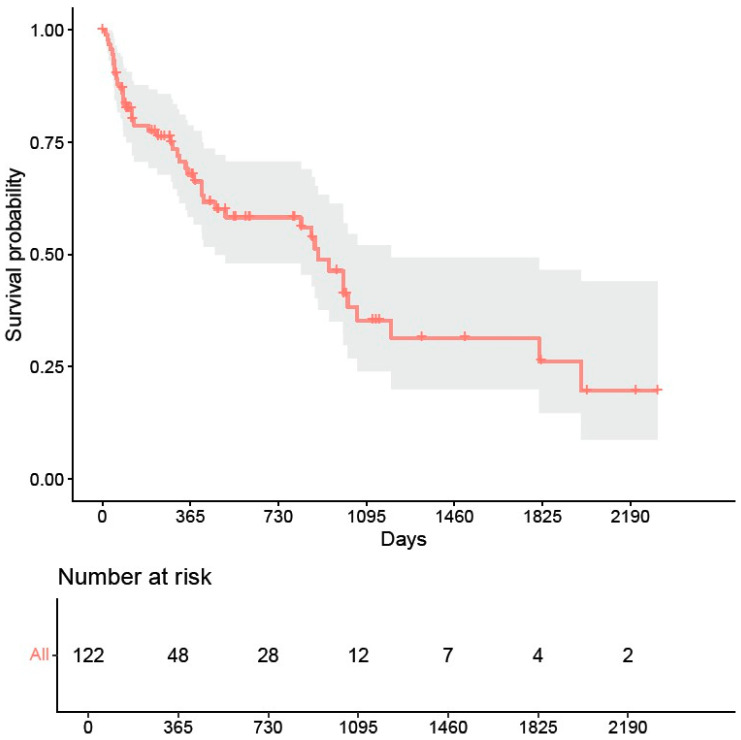
Kaplan–Meier analysis for the time between selective laser trabeculoplasty and subsequent surgical intervention.

At 365 days (1 year) after selective laser trabeculoplasty, no further pressure-reducing intervention was necessary in 67.8% of the eyes. After 730 days (2 years), 58.3% of the eyes remained without a subsequent intervention (Figure 1).

**Figure 2:**
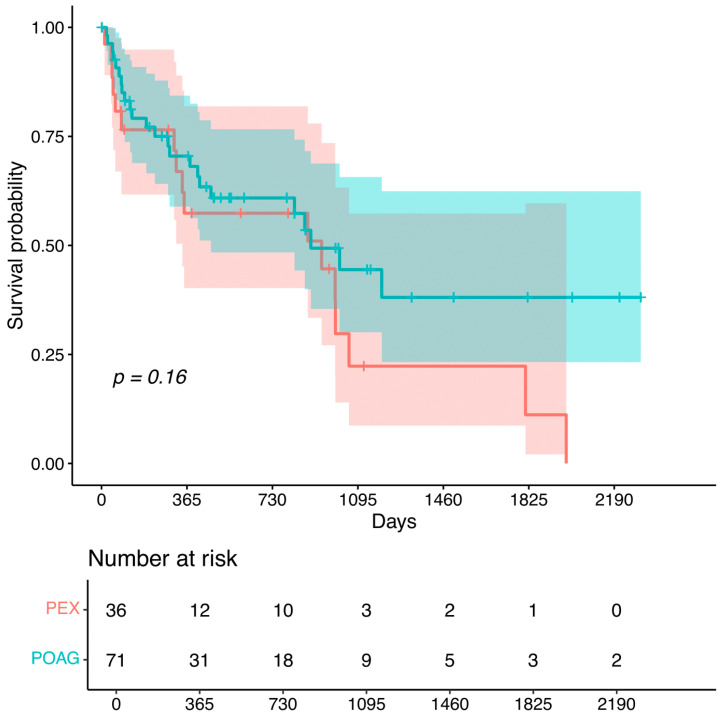
Kaplan–Meier analysis for the time between selective laser trabeculoplasty and subsequent surgical intervention in patients with pseudo exfoliation glaucoma (PEX) or primary open-angle glaucoma (POAG).

In patients with primary open-angle glaucoma (POAG), 365 days (1 year) after selective laser trabeculoplasty, no further pressure-reducing intervention was needed in 70.5% of the eyes. After 730 (2 years) days, 60.9% of the eyes remained without a subsequent intervention. In patients with pseudo exfoliation glaucoma (PEX), 365 and 730 days (1 to 2 years) after selective laser trabeculoplasty, no further pressure-reducing intervention was necessary for 57.4% of the eyes (Figure 2).

A Cox regression model was utilized to identify risk factors for subsequent interventions during the follow-up period. The factors examined included age, gender, preoperative intraocular pressure (IOP), glaucoma type, and the number of interventions prior to selective laser trabeculoplasty (SLT). The baseline IOP demonstrated a statistically significant impact on the likelihood of requiring further intervention (*p* = 0.00561). All other parameters did not achieve statistical significance at the 5% level.

## 4. Discussion

The results of our study provide an insight into the efficacy and effect duration of selective laser trabeculoplasty (SLT) following prior pressure-reducing interventions in glaucoma patients with a specific focus on the comparison between primary open-angle glaucoma (POAG) and pseudo exfoliation glaucoma (PEX). We observed that after 365 days (1 year), approximately 68% of the eyes did not require any additional pressure-lowering surgery. After 730 days (2 years), this percentage decreased to 58%.

There was a trend indicating that patients with pseudo exfoliation glaucoma were more likely to undergo a secondary pressure-lowering intervention earlier than those with POAG.

The effect of SLT after trabeculotomy or comparable anterior chamber angle procedures is presumably reduced because the trabecular meshwork (TM) is opened over multiple clock hours, making effective laser treatment difficult. We assume that SLT in patients with prior anterior chamber angle surgical intervention likely results in a differential pressure-lowering effect.

### 4.1. Longevity of the SLT Efficacy

The Kaplan–Meier survival analysis indicates that, across the entire cohort, SLT is an effective intermediate intervention, with 67.8% of eyes remaining free from further pressure-reducing procedures after one year. This figure decreases to 58.3% after two years, illustrating a gradual decline in the efficacy of SLT over time. This reduction aligns with previous studies, which have documented a decrease in SLT effectiveness over time for treatment-naïve eyes and eyes with prior pressure-lowering surgeries [1,2]. The observed decline suggests that while SLT is beneficial in delaying subsequent interventions, its effects are not permanent, necessitating eventual additional treatments [2,12].

Due to the small number of patients still being monitored by us after 3 years, we cannot provide a reliable long-term prognosis for the effectiveness of SLT following a previous glaucoma procedure. However, as the pressure-lowering effect of SLT diminishes over time, in most cases, supplementary therapy is necessary in the medium to long term [13,14].

Current research consistently supports the use of SLT as a viable option to delay additional medications or more invasive glaucoma surgeries [2,5]. However, this study adds to the body of evidence by highlighting the variability in SLT efficacy based on the glaucoma subtype. To the best of our knowledge, there are not many studies comparing the outcome of SLT for PEX glaucoma and POAG, especially not after a prior pressure-lowering procedure. Koucheki et al. did not find a significant IOP difference in the post-SLT measurements comparing PEX glaucoma and POAG in patients without any prior pressure-lowering surgery [8]. Thus, our findings are consistent with previous studies that have noted the more aggressive course of PEX with, e.g., more rapid visual field loss, which often requires more intensive management strategies [7].

### 4.2. Differential Outcomes in POAG vs. PEX

The study’s specific breakdown of outcomes for POAG and PEX patients reveals some differences. In POAG patients, 70.5% did not require additional interventions after one year, dropping to 60.9% after two years. In contrast, PEX patients exhibited lower rates of remaining intervention-free, with 57.4% at both one and two years. This consistent necessity for additional interventions in PEX patients emphasizes the more aggressive nature of PEX glaucoma [15] and its resistance to sustained control by SLT alone. Although we observed a tendency for patients with PEX to undergo secondary treatment earlier compared to those with POAG, research on other angle-closure surgeries suggests that there is no significant difference between PEX and POAG regarding the IOP-lowering effect [7,8]. Pahitzsch et al. found that the time to additional surgery for patients with PEX following ab interno trabeculectomy was shorter than for those with POAG, although their findings were not significant [9]. In a study by Zhou et al., patients with PEX glaucoma or POAG exhibited a failure rate of 84% after 2 years for non-operated patients in both groups. Interestingly, patients with steroid-induced glaucoma demonstrated a better response compared to those with PEX or POAG [16].

### 4.3. Limitations

The limitations of this study include its retrospective design and the potential for loss to follow-up. However, given the large catchment area of our center, we assume that patients requiring a following pressure-lowering intervention would have likely returned to our clinic for this procedure.

## 5. Conclusions

The study’s findings on the effectiveness and duration of SLT following prior pressure-reducing interventions highlight differences in outcomes between POAG and PEX patients. While SLT remains a valuable tool in the glaucoma treatment arsenal for eyes with prior pressure-lowering interventions, particularly for POAG patients, the need for tailored treatment plans and vigilant follow-up is evident. These results reinforce the importance of considering individual patient characteristics and disease progression patterns in the management of glaucoma to optimize therapeutic outcomes. Further research is essential to refine SLT protocols and explore additional or alternative treatments that could enhance long-term efficacy, particularly for PEX patients.

## Figures and Tables

**Table 1:** OPS codes for the analyzed pressure-reducing procedures.

OPS Code	Procedure
5-131 (0.0, 0.00, 0.01, 0.0x)	Filtration Surgery
5-131.6 (0,1,2,3x)	(Filtration) Surgery with implant
5-131.7 (5-133-1, 5-133.8 (0,1))	Trabeculotomy (laser, electroablation)
5-132.2 (0,1,2,x, 5-132.x,y)	Cyclophotocoagulation

**Table 2:** Descriptive statistics.

Data	Mean (*n* = 122)
Age of patients (in years)	73.86 +/− 10.11
Sex (female)	57%
Procedures before SLT	1.53
Baseline pressure (in mmHg)	20.29 +/− 4.81
IOP ^1^ after SLT in patients that needed surgical intervention (in mmHg)	24.1 +/− 6.46
Pressure-reducing medications prior SLT	2.54 +/− 1.28
Pressure-reducing medications after SLT in patients that needed surgical intervention	2.56 +/− 1.52

^1^ IOP = Intraocular pressure.

**Table 3:** Type of glaucoma.

Type	Number (*n* = 122)
Primary open-angle glaucoma	71
Pseudoexfoliation glaucoma	36
Myopia-related glaucoma	3
Normal-tension glaucoma	2
Pigmentary glaucoma	2
Angle-closure glaucoma	2
Dysgenetic glaucoma	1
Other ^1^	5

^1^ Other: rubeotic secondary glaucoma, uveitis, unknown.

## Data Availability

Data are available on reasonable request.
